# Brain perfusion CT compared with ^15^O-H_2_O PET in patients with primary brain tumours

**DOI:** 10.1007/s00259-012-2173-1

**Published:** 2012-06-27

**Authors:** Julie Marie Grüner, Rune Paamand, Michael Kosteljanetz, Helle Broholm, Liselotte Højgaard, Ian Law

**Affiliations:** 1 Department of Clinical Physiology, Nuclear Medicine and PET, Rigshospitalet, University of Copenhagen, Blegdamsvej 9, 2100 Copenhagen, Denmark; 2 Department of Neurosurgery, Rigshospitalet, University of Copenhagen, Blegdamsvej 9, 2100 Copenhagen, Denmark; 3 Department of Neuropathology, Rigshospitalet, University of Copenhagen, Blegdamsvej 9, 2100 Copenhagen, Denmark

**Keywords:** PET, Brain perfusion, Brain tumour, Oxygen radioisotopes, Perfusion CT

## Abstract

**Purpose:**

Perfusion CT (PCT) measurements of regional cerebral blood flow (rCBF) have been proposed as a fast and easy method for identifying angiogenically active tumours. In this study, quantitative PCT rCBF measurements in patients with brain tumours were compared to the gold standard PET rCBF with ^15^O-labelled water (^15^O-H_2_O).

**Methods:**

On the same day within a few hours, rCBF was measured in ten adult patients with treatment-naïve primary brain tumours, twice using ^15^O-H_2_O PET and once with PCT performed over the central part of the tumour. Matching rCBF values in tumour and contralateral healthy regions of interest were compared.

**Results:**

PCT overestimated intratumoural blood flow in all patients with volume-weighted mean rCBF values of 28.2 ± 18.8 ml min^−1^ 100 ml^−1^ for PET and 78.9 ± 41.8 ml min^−1^ 100 ml^−1^ for PCT. There was a significant method by tumour grade interaction with a significant tumour grade rCBF difference for PCT of 32.9 ± 15.8 ml min^−1^ 100 ml^−1^ for low-grade (WHO I + II) and 81.5 ± 15.4 ml min^−1^ 100 ml^−1^ for high-grade (WHO III + IV) tumours, but not for PET. The rCBF PCT and PET correlation was only significant within tumours in two patients.

**Conclusion:**

Although intratumoural blood flow measured by PCT may add valuable information on tumour grade, the method cannot substitute quantitative measurements of blood flow by PET and ^15^O-H_2_O PET in brain tumours.

## Introduction

The recent technical developments in fast multidetector CT and commercial software solutions have enabled a fast, relatively simple, practical and available approach to assess essential parameters of vascular physiology, namely regional cerebral blood flow (rCBF), regional cerebral blood volume (rCBV) and the permeability-surface area product (PS)—a measure of blood-brain barrier permeability [1]. This technique, known as perfusion CT (PCT), has primarily found routine clinical use in the management of acute ischaemic stroke and is considered a valid alternative to perfusion-weighted MRI (PWI)[1, 2]. In oncology there is a growing interest in methods for evaluation of tumour-associated neovasculature. This process, known as angiogenesis, is induced early during the multistage development of invasive cancers and has long been recognized as one of the important integral hallmarks of cancer [3]. PCT has clinical value in neuro-oncology and can contribute to the preoperative grading of primary brain tumours [4]. An effective option would be to add PCT to molecular imaging studies already being performed in the clinical workup of brain tumour patients using hybrid PET/CT scanning systems. If equipped with state-of-the-art multislice CT scanners with 40–128 detector rows that, using constant periodic bidirectional table movements, can cover 96 mm of the brain in the z-axis [4], it would extend the utility of CT to provide further clinical information—not just low-dose CT for attenuation correction, thus mimicking the multiparametric approach that is now the standard strategy using MRI. PCT might thereby give a fingerprint of the tumour microenvironment combining vascular physiological measures with radioisotope images of tissue hypoxia [5], amino acid transport, tumour proliferation [6] or glucose metabolism [7] in a single session. This opportunity for studying the metabolism/perfusion relationship with PET/PCT had been recognized early on [1], but is rarely utilized [8, 9].

The precondition, however, is that PCT measurements obtained in a tumour using standard methods are robust and reliable and relate genuinely quantitative physiological parameters. In a previous independent study, using healthy subjects, we found this not to be the case. There was a significant overestimation of rCBF by PCT in grey matter of 47 % on average, validated against a recognized gold standard, PET with ^15^O-labelled water [10].

The aim of this study was to compare PCT to the gold standard ^15^O-H_2_O PET for brain perfusion, both calculated with a deconvolution approach in a prospective study of the two techniques. These assessments were performed directly preoperatively in patients with primary brain tumours, on the same day, within a few hours.

## Materials and methods

### Patients

Ten adult patients (four women and six men) with a median age of 53 years (range 24–63 years) were recruited prospectively and scanned from October 2008 to December 2010. The inclusion criteria were patients with initial untreated presentations of suspected primary supratentorial brain tumours based on the MRI findings. The exclusion criteria were systemic malignancies, reduced renal function, pregnancy, biguanide-treated diabetes and known allergy to iodinated contrast media. The plasma creatinine levels of all patients were measured within 2 weeks before the scan and were all within the normal range. The protocol was approved by the Committees on Biomedical Research Ethics for the Capital Region of Denmark (protocol number H-A-2008-055). All patients gave oral and written informed consent according to the Helsinki II Declaration. Nine of the patients had intra-axial tumours and one had an extra-axial tumour (meningioma). All patients with high-grade glioma (WHO III and IV) with the exception of one (*n* = 4) were treated with corticosteroids before and during PET and CT scanning. Antiepileptic drugs were administered to six patients. Tumour tissue was biopsied a median of 12 days (range 3–453 days) after PET and CT scanning perioperatively and histologically verified and graded according to the WHO criteria by an experienced neuropathologist (H.B.). The patients and their tumours are characterized in Table 1.

**Table 1:** Patient characteristics

Patient	Age	Sex	Tumour type	WHO grade	Side	Lobe	Measure	Prednisone	AED	CE	Appearance
1	32	M	Astrocytoma	II	R	T	5 × 9 × 3	−	−	−	Solid
**2**	**64**	**M**	**Glioblastoma multiforme**	**IV**	**L**	**T**	**4 ×** **5 ×** **4**	**+**	**+**	**+**	**Cystic**
3	55	F	Astrocytoma	II	R	F	5 × 6 × 5	+	+	+	Solid
4	53	F	Neuroepithelial tumour	II	R	P	3 × 3 × 3	−	+	−	Solid
**5**	**63**	**M**	**Glioblastoma multiforme**	**IV**	**L**	**T**	**5 ×** **4 × 4**	**+**	**−**	**+**	**Cystic/solid**
6	46	F	Meningioma	I	L	F	4 × 5 × 5	+	+	+	Cystic/solid
**7**	**56**	**M**	**Glioblastoma multiforme**	**IV**	**R**	**F**	**5 × 5** **× 3**	**+**	**−**	**+**	**Cystic/solid**
**8**	**30**	**M**	**Anaplastic oligodendroglioma**	**III**	**R**	**P**	**2 × 2** **× 2**	**−**	**+**	**+**	**Cystic/solid**
9	24	F	Astrocytoma	II	L	F	4 × 5 × 4	−	+	+	Cystic/solid
**10**	**55**	**M**	**Glioblastoma multiforme**	**IV**	**R**	**F**	**5 × 5** **× 6**	**+**	**−**	**+**	**Cystic/solid**

Tumour type and tumour grade listed according to the WHO classification. Measures are in centimetres, side-to-side, anteroposterior, craniocaudal to the nearest centimetre. High-grade tumours are marked in **bold**

*AED* antiepileptic drugs, *CE* contrast enhancement in the tumour with T1-weighted MRI scan, *F* frontal, *T* temporal, *P* parietal

### Imaging

All patients had pre- and post-contrast MRI scans as part of the routine preoperative workup including standard T1-weighted imaging with and without gadolinium-based contrast, T2-weighted imaging and fluid-attenuated inversion recovery (FLAIR). This was performed a median of 19 days before rCBF PET and CT scanning (range 8–110 days).

### PET protocol

#### Scanner

A dedicated brain HRRT (High Resolution Research Tomograph) PET scanner (CTI/Siemens, Knoxville, TN, USA) was used for all ^15^O-H_2_O PET scans. This scanner has an axial field of view of 25 cm and a near isotropic resolution of 2 mm.

#### PET tracer

For the scan, 800 MBq ^15^O-H_2_O was produced online and injected intravenously into an antecubital vein via an Automatic Water Injection System (AWIS 1997, Scansys by Peter Larsen, Denmark). AWIS delivered a 16-ml bolus over 10 s with both pre-flush and after-flush of an inert saline solution [11].

Nine patients received two tracer injections, and one patient had only one tracer injection because of clotting in the arterial catheter after the first scan. A short indwelling catheter was placed in the non-dominant radial artery under local anaesthesia for blood sampling.

#### Image acquisition

During scanning, the patient’s head rested in a foam-cushioned headrest, and a head strap was used to minimize head movement. Initially a 6-min transmission scan with a rotating ^137^Cs single photon point source was performed for attenuation correction. The 7-min emission scans were acquired in 3D list mode and initiated immediately before tracer injection. The interscan interval was at least 10 min to allow for isotope decay.

For kinetic modelling arterial blood was sampled continuously during the scans using an automatic blood sampling system (Allogg ABSS, Mariefred, Sweden) set to draw arterial blood at a constant speed of 8 ml/min with activity measures every 0.5 s. The inner diameter of the tube connected to the arterial catheter was 1.0 mm. The detectors in the ABSS and the PET scanner were cross-calibrated against an independent dose calibrator so that all data could be reported in radioactivity concentration (Bq/ml). Immediately after the scan, 2 ml arterial blood was drawn for blood gas analysis to evaluate the physiological respiratory state of the patient. The samples were analysed for arterial partial pressure of oxygen and carbon dioxide (*P*_a_O_2_, *P*_a_CO_2_), saturation level of oxygen (sO_2_) and haemoglobin concentration (ctHb) (ABL 700 Series, Radiometer Medical, Copenhagen, Denmark).

#### Image reconstruction

Dynamic images were reconstructed using a 3D ordered subset expectation maximization algorithm with correction for the measured point spread function (3D-OSEM-PSF) into 40 frames per scan of 1 × 30, 18 × 5, 9 × 10, 10 × 15 and 2 × 30 s durations. Each frame consisted of 207 image planes in a 256 × 256 matrix with an isotropic voxel size of 1.22 × 1.22 × 1.22 mm^3^. The first 30-s frame was designed to accommodate the tracer delay from injection to brain tissue. All images were corrected for randoms, scatter, attenuation, decay and dead time and filtered with a 3D Gaussian 5-mm filter.

#### PET CBF calculation

Using a commercially available software package, PMOD 3.0 (PMOD Technologies, Zürich, Switzerland), the dynamic images of the first 210 s following arrival of activity to the brain and the delay and dispersion-corrected arterial input function [12] were fitted by a standard one-tissue compartment model (“Alpert’s one-tissue compartment model”) [13, 14] according to Eq. 1.

1 $$ {C_t}(t) = f{C_a}(t) \otimes {e^{{ - \left( {{{f} \left/ {{{V_T}}} \right.}} \right)t}}} $$

where *C*(*t*) denotes tissue activity concentration (Bq/ml), *Ca*(*t*) the measured arterial input function (Bq/ml), *f* is rCBF (ml min^−1^ 100 ml) and *V*_T_ (ml/g) is the fitted volume of distribution. ⊗ represents the convolution operation. This generated parametric images of rCBF.

#### Radiation dose

The dose equivalent following PET transmission and emission scans was in total 1.6 mSv, 0.1 mSv for the transmission scan and 0.74 mSv for each emission scan.

### CT protocol

#### Scanner

Biograph 40 TruePoint PET/CT scanner (Siemens, Knoxville, TN, USA) with a 40 detector row CT was used.

#### Contrast media

A preheated iso-osmolar iodine contrast medium OptiRay 350 (Ioversol 350 mg/ml, Tyco Healthcare, Neustadt/Donau, Germany) was injected intravenously by a power injector (OptiVantage DH Injection System, Liebel-Flarsheim, Cincinnati, OH, USA) as a short bolus of 40 ml (8 ml/s) through a catheter in the antecubital vein followed by 20 ml saline solution.

#### Image acquisition

The PET scanning was, for practical reasons, performed in all patients before CT on the same day and within 1–4 h. A lateral scout scan at the angle of the meato-orbital plane was performed followed by a non-enhanced low-dose CT (120 kVp, 40 mAs). The selection of four contiguous transaxial slices with a coverage of 28 mm through the centre of the tumour was guided with reference to the corresponding contrast-enhancing T1-weighted MRI or T2-weighted MRI in non-enhancing tumours. This was done either visually or by direct MRI to low-dose CT coregistration on a separate workstation next to the CT acquisition computer. The orbital fossa was avoided in order to reduce irradiation to the lens of the eye. The contrast medium was injected 4 s before initiation of the dynamic scan. The dynamic scan consisted of 160 images: one image/s for 40 s over four slices 7.2 mm thick at 80 kVp, 200 mAs for patients 1-6 and 80 kVp, 120 mAs for patients 7-10. The arterial cannula from the PET scan was kept in place and used to draw arterial blood for blood gas analysis immediately after the scan.

#### Image reconstruction and rCBF calculation

Each slice was reconstructed into a 512 × 512 image matrix using an H30s medium smooth kernel. Voxel dimensions were non-isotropic 0.44 × 0.44 × 7.2 mm^3^. The rCBF was calculated in a semi-automated manner using commercial software, Syngo Neuro Perfusion CT 2006A (Siemens). After segmentation and removal of extra-cerebral tissue, a circular reference region of interest (ROI) was defined automatically in the occipital part of the superior sagittal sinus. Maximum intensity projections (MIP-CT) were reconstructed to enhance areas of high radiodensity that are useful for identifying vascular structures. The larger vessels were removed by thresholding the MIP-CT image by 15 %. The arterial input function was derived from the time-attenuation curve from ROIs comprising either anterior cerebral arteries in cross section or medial cerebral arteries, or both, and the rCBF was calculated using a deconvolution approach [15].

#### Radiation dose

The effective dose equivalent was 2.9 mSv for the CT protocol at 120 mAs and 3.8 mSv at 200 mAs.

### Data analysis

#### Image coregistration and regions of interest

**Fig. 1:**
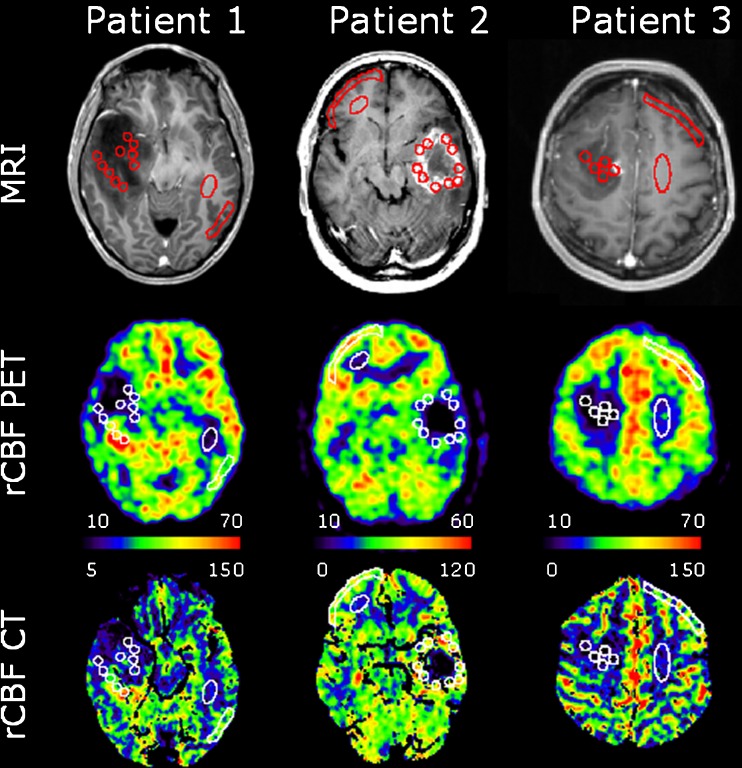
Patients with coregistered transaxial slices of post-contrast preoperative T1-weighted MRI, rCBF PET and PCT scans (ml min^−1^ 100 ml^−1^) documenting the locations of ROIs. Highly vascular areas are eliminated in PCT. Patient 1 suffered from a heterogeneous, non-enhancing astrocytoma (grade II) with central hypoperfused and posterior hyperperfused areas. Patient 2 had a cystic ring-enhancing moderately perfused glioblastoma multiforme (grade IV). Patient 3 had an enhancing uniformly hypoperfused astrocytoma (grade II). Note the different scaling of rCBF PET and rCBF CT images. The *top row* shows T1-weighted MR images with gadolinium contrast

**Fig. 2:**
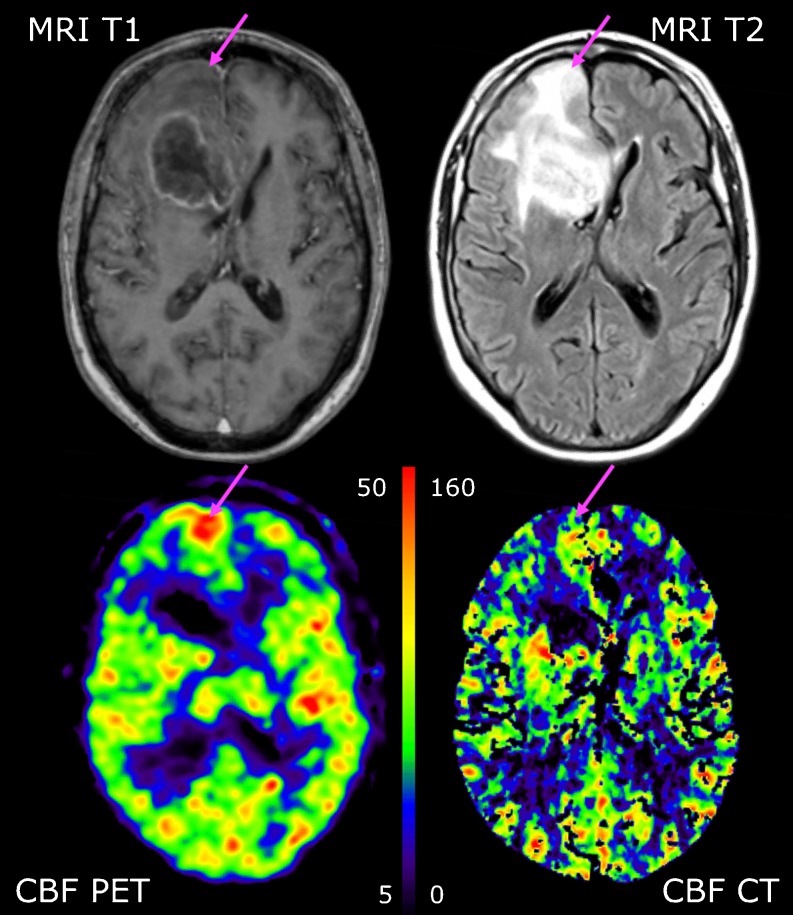
Coregistered transaxial slices of post-contrast preoperative T1-weighted MRI and FLAIR sequences (*top row*) performed 15 days prior to rCBF PET and rCBF CT scans (*bottom row*) in patient 7, suffering from glioblastoma multiforme (grade IV). The tumour has a hypoperfused cystic necrotic centre and heterogeneous hypo- and hyperperfusion in the contrast-enhancing wall. PET shows a hyperperfused area anterior to the tumour (*arrow*) that cannot be identified on rCBF CT performed within 2 h. This is likely to represent a transient non-convulsive epileptic seizure. rCBF is measured in ml min^−1^ 100 ml^−1^

Using PMOD, rCBF PET, rCBF CT and MRI images were coregistered to the low-dose CT scan of the head to a final voxel size of 0.5 × 0.5 × 1.5 mm^3^. This was done to ensure that all ROIs referred to and included an identical tissue composition throughout all techniques. On the MRI images, ten non-overlapping and independent circular ROIs with a diameter of 7.2 mm and a sample volume of 61 mm^3^ were drawn, an irregular freehand ROI in normal appearing contralateral grey matter and an ellipsoid in normal appearing contralateral white matter. The tumour ROIs were, as far as possible, placed outside highly vascular regions. This, however, was difficult, particularly in the high-grade glioma. Vascular voxels within an ROI with an rCBV larger than 15 ml/100 g were excluded from analysis by a masking technique. In patient 5, suffering from a cystic glioblastoma multiforme with a thin highly vascularized tissue rim, only six ROIs could be confidently placed. Finally, the ROIs were projected onto identical areas of the masked parametric rCBF PET and rCBF CT images for quantification (Figs. 1 and 2). The tissue volume in the tumour ROIs ranged from 30 to 63 mm^3^ (mean 54 mm^3^), in white matter from 123 to 357 mm^3^ (mean 228 mm^3^) and in grey matter from 321 to 938 mm^3^ (mean 572 mm^3^). We then calculated the volume-weighted average tumour values based on the ROIs.

### Statistical methods

The statistical analysis was conducted using MATLAB (MathWorks Inc., Natick, MA, USA). Paired *t* tests with a two-tailed significance level of α = 0.05 were used to test for differences in the measures of blood gas data. A similar method was used for the analyses between rCBF CT and the average of two rCBF PET scans within each patient for every tumour, and between patients for the volume-weighted tumour rCBF, and for the white and grey matter rCBF. In the clinical use of PCT and MRI it is recognized that the global variation may significantly influence the regional variation. We therefore normalized the tumour and grey matter rCBF to the normal appearing white matter rCBF and repeated the statistical analysis.

The Bland-Altman test was used to assess the agreement between corresponding tumour measurements. The mean difference, standard deviations and the 95 % limits of agreement were calculated and plotted. For tumours the intra-patient and between-patient correlation of the two methods was calculated using linear regression analyses as well as the coefficient of determination (*r* ^2^) between methods.

For both absolute and white matter normalized values we used a two-way analysis of variance (ANOVA) for modelling the interaction effect and the main effects of two factors, namely imaging technique (PET or PCT) and tumour grade [low (WHO grade I and II) or high (WHO grade III and IV)]. Also, we performed unpaired *t* tests with a two-tailed significance level of α = 0.05 to test for differences between high- and low-grade tumours for both absolute and white matter normalized rCBF values, for PET and PCT separately. These analyses comprised only the glioma with exclusion of one outlier, patient 9 with grade II astrocytoma (Table 1) and unusually high tumour tissue perfusion on PET and PCT.

## Results

### Blood gas analyses

Eight patients were included in the blood gas analysis (Table 2). For the two excluded patients, the arterial cannula malfunctioned before arterial blood samples were drawn. The ctHb and sO_2_ did not change significantly between the PET and PCT studies. The *P*_a_CO_2_, however, dropped significantly by 0.5 kPa, or 4 mmHg, and the *P*_a_O_2_ increased by 1.9 kPa or 14 mmHg from PET scan to PCT scan. This indicates hyperventilation during performance of PCT.

**Table 2:** Blood gas measurements (*n* = 8)

	rCBF PET	rCBF CT	*p* value
*P*_a_CO_2_ (kPa)	5.6 ± 0.59	5.1 ± 0.57	< 0.05
*P*_a_O_2_ (kPa)	11.8 ± 1.47	13.7 ± 1.83	< 0.05
ctHb (mmol/l)	8.5 ± 1.41	7.5 ± 1.72	NS
sO_2_ (%)	0.96 ± 0.01	0.97 ± 0.05	NS

*P*_*a*_ *CO*_*2*_ arterial partial pressure of carbon dioxide, *P*_*a*_ *O*_*2*_ arterial partial pressure of oxygen, *ctHb* haemoglobin concentration, *sO*_*2*_ oxygen saturation, all listed as mean ± SD

### Regional cerebral blood flow measures

The analysed mean volume of the tumours was 0.52 ± 0.11 ml (data not shown). In tumours, volume-weighted mean rCBF values were 28.2 ±18.8 (range 12.1–73.2) ml min^−1^ 100 ml^−1^ for PET and 78.9 ± 41.8 (range 14.7–142.2) ml min^−1^ 100 ml^−1^ for PCT (Table 3). In all but one patient, PET and PCT values differed significantly. The *r* ^2^ ranged from 0.02 to 0.91, mean 0.22, and three patients had significant intratumoural correlation between PET and PCT values. Using PET, mean tumour blood flow was not significantly different between low (I–II) or high (III–IV) WHO graded gliomas measured at 18.4 ± 8.3 and 21.5 ± 7.0 ml min^−1^ 100 ml^−1^, respectively (Table 4). In PCT, however, there was a significant tumour grade difference (*p* <0.01) in perfusion with values of 32.9 ± 15.8 and 81.5 ± 15.4 ml min^−1^ 100 ml^−1^ for low and high grade, respectively. Both grey and white matter values were extracted from the patients’ scans (Table 3). The mean grey matter value was 34.9 ± 8.8 ml min^−1^ 100 ml^−1^ in PET and 74.6 ± 13.7 ml min^−1^ 100 ml^−1^ in PCT, and the mean white matter value was 15.9 ± 4.8 ml min^−1^ 100 ml^−1^ in PET and 29.7 ± 3.9 ml min^−1^ 100 ml^−1^ in PCT. These differences were significant.

**Table 3:** rCBF measurements from PET and CT scanning

Patient	Tumour			Grey matter		White matter		Tumour normalized		Grey matter normalized	
	PET	CT	*r* ^2^	PET	CT	PET	CT	PET	CT	PET	CT
1	27.8 ± 16.1	43.4 ± 32.8*	0.91**	32.9	70.9	16.5	34.1	1.7 ± 1.0	1.3 ± 1.0*	2.0	2.1
**2**	**29.2 ±** **5.2**	**71.0 ±** 9.9*	**0.06**	**49.7**	**64.7**	**20.7**	**24.5**	**1.4 ±** **0.3**	**2.9 ±** 0.4*	**2.4**	**2.6**
3	12.1 ± 1.3	14.7 ± 4.8	0.08	45.9	58.5	21.8	26.4	0.6 ± 0.1	0.6 ± 0.2	2.1	2.2
4	15.4 ± 1.7	40.5 ± 10.1*	0.05	27.9	81.5	12.2	29.5	1.3 ± 0.1	1.4 ± 0.3	2.3	2.8
**5**	**12.4 ±** **4.8**	**96.2 ±** 22.0*	**0.14**	**20.9**	**81.7**	**11.3**	**36.6**	**1.1 ±** **0.4**	**2.6 ±** 0.6*	**1.8**	**2.2**
6	45.9 ± 20.1	142.2 ± 53.8*	0.55**	35.1	62.7	20.7	29.9	2.2 ± 1.0	4.8 ± 1.8*	1.7	2.1
**7**	**27.5 ±** **12.3**	**82.6 ±** 18.4*	**0.02**	**32.5**	**77.9**	**11.7**	**26.9**	**2.3 ±** **1.1**	**3.1 ±** **0.7**	**2.8**	**2.9**
**8**	**17.1 ±** **2.8**	**61.4 ±** 19.8*	**0.03**	**32.8**	**101.2**	**11.4**	**32.0**	**1.5 ±** **0.2**	**1.9 ±** **0.6**	**2.9**	**3.2**
9	73.2 ± 19.3	141.0 ± 32.2*	0.08	42.7	60.2	21.6	25.7	3.4 ± 0.9	5.5 ± 1.3*	2.0	2.3
**10**	**20.9 ±** **6.8**	**96.3 ±** 36.8*	0.31**	**28.9**	**86.7**	**11.4**	**31.4**	**1.8 ±** **0.6**	**3.1 ±** 1.2*	**2.5**	**2.8**
Mean ± SD	28.2 ± 18.8	78.9 ± 41.8***	0.22	34.9 ± 8.8	74.6 ± 13.7***	15.9 ± 4.8	29.7 ± 3.9***	1.7 ± 0.8	2.7 ± 1.5***	2.2 ± 0.4	2.5 ± 0.4***

Tumour, grey matter and white matter ROIs, and tumour ROIs and grey matter ROIs normalized with white matter, listed as mean ± SD. Volume-weighted rCBF is given in ml min^−1^ 100 ml^−1^. High-grade tumours are marked in **bold**

*Significant difference between modalities in the individual patient, *p* <0.05

**Significant correlation between modalities in the individual patient, *p* <0.05

***Significant difference between modalities in all patients, *p* <0.05

**Table 4:** rCBF measurements from PET and CT scanning (*n* = 8)

	Tumour			Tumour normalized	
	PET	CT	*r* ^2^	PET	CT
Total low-grade tumours	18.4 ± 8.3	32.9 ± 15.8	0.35 ± 0.5	1.2 ± 0.4	1.1 ± 0.4
Total high-grade tumours	21.5 ± 7.0	81.5 ± 15.4	0.11 ± 0.1	1.6 ± 0.5	2.7 ± 0.5

Tumour tissue perfusion in absolute measures and normalized to white matter, stratified according to WHO classification (Table 1), and listed as mean ± SD. Low grade: I-II (patients 1, 3, 4), high grade: III-IV (patients 2, 5, 7, 8, 10). Patient 6 excluded because of tumour type (meningioma). Patient 9 excluded as an outlier

Blood flow is given in ml min^−1^ 100 ml^−1^. Ratios are volume weighted

Mean ratios of tumour rCBF over white matter rCBF were 1.7 ± 0.8 and 2.7 ± 1.5 for PET and PCT, respectively, including all patients. This quite large difference in normalized rCBF values between modalities was significant for six of ten patients. The mean ratio of grey over white matter was 2.2 ± 0.4 for PET and 2.5 ± 0.4 for PCT. This difference between modalities was also significant. When tumour perfusion was normalized to white matter there was only a significant difference between tumour grade for PCT (*p* < 0.005; low grade 1.1 ± 0.4; high grade 2.7 ± 0.5), but not for PET (low grade 1.2 ± 0.4; high grade 1.6 ± 0.5; Table 4).

**Fig. 3:**
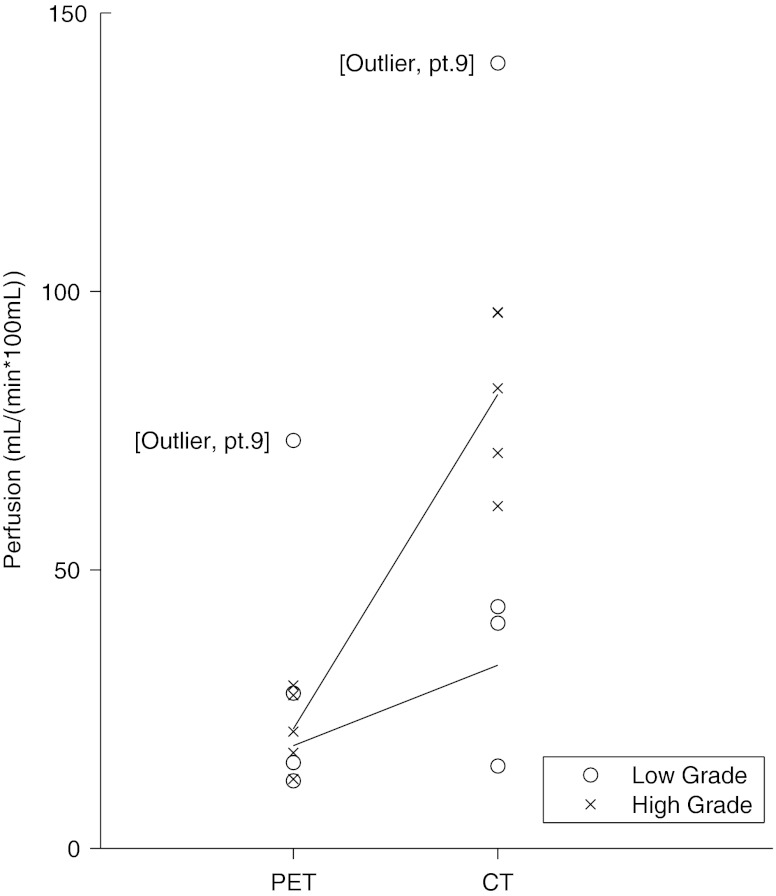
Comparison of rCBF PET and PCT by glioma grade. The perfusion measuring technique interacts significantly with tumour grade. rCBF for tumour grade is only significantly different when measured with PCT. o: low-grade glioma, x: high-grade glioma, *n* = 8

The ANOVA of absolute and white matter normalized tumour perfusion both revealed a significant main effect of technique (PCT > PET), a significant main effect of grade (high grade > low grade) and a significant interaction effect (technique × grade) (Fig. 3). The grade and the interaction effects are likely to be driven by the high rCBF in high-grade tumours measured with PCT, as the difference between grades only were significant using PCT, not using PET.

**Fig. 4:**
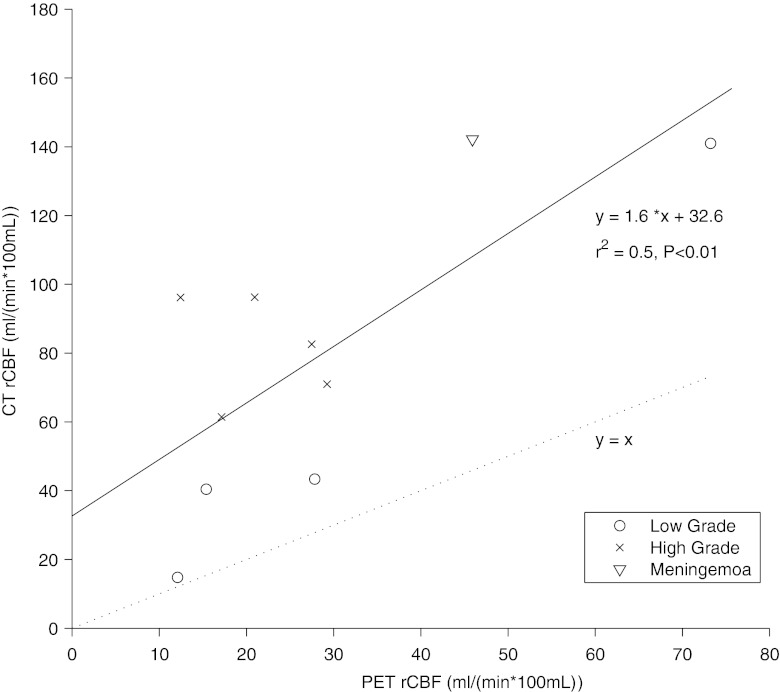
The within-patient correlation of PET and PCT measurements. o: low-grade glioma, x: high-grade glioma, ▽ : meningioma, *n* = 10

**Fig. 5:**
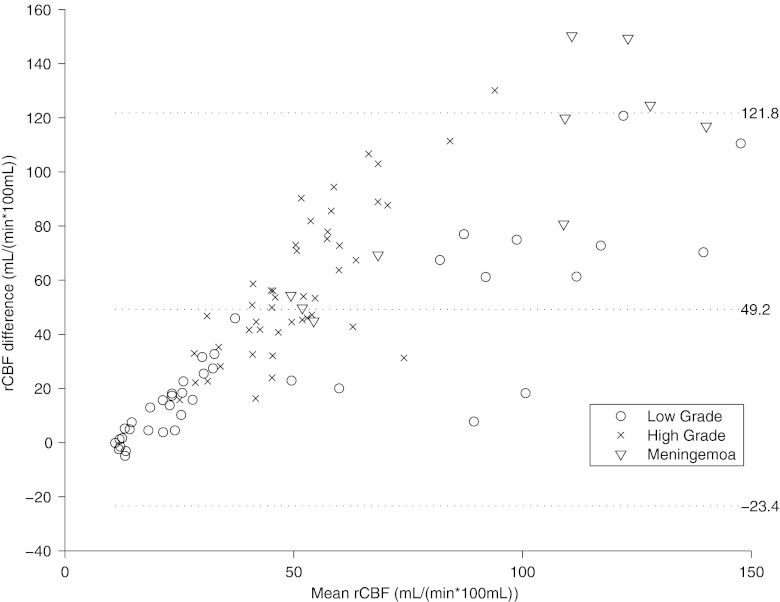
Bland-Altman plot. The *middle line* indicates the mean difference. The *outer lines* indicate 95 % limits of agreement. The differences between rCBF PET and PCT increase with increasing rCBF values. o: low-grade glioma, x: high-grade glioma, ▽ : meningioma, *n* = 10

The within-patient correlation of rCBF between modalities was significant for three patients (Table 3). The between-subject whole tumour rCBF correlation was significant when using absolute values (*r* ^2^ = 0.5, slope 1.6; intercept 32.6 ml min^−1^ 100 ml^−1^; Fig. 4), and even better when values normalized to the white matter were used (*r* ^2^ = 0.7; slope 1.6; intercept 1.0). A Bland-Altman plot over each individual ROI shows bias for PCT rCBF values, which increases with increasing tumour rCBF values (Fig. 5), and the bias is similar for the three tumour types mentioned in this study.

## Discussion

In this study, our objective was to test the validity of the preoperative quantitative measurements of rCBF in ten patients with brain tumours using PCT, a novel and promising technique, against the gold standard ^15^O-H_2_O PET [16] (Table 1). We found that PCT overestimated gliomatous tumour rCBF values compared to ^15^O-H_2_O PET by 22–672 %.

First, we examined both absolute quantified rCBF values and rCBF values normalized to healthy appearing white matter in the hemisphere contralateral to the malignancy for both methods, PCT and PET. Normalization is used as a standard method in the evaluation of tumours in MRI [17, 18] and PCT [19] to increase the robustness of the regional physiological measures (rCBV, rCBF) by removing global variation influenced by particular aspects of the technique, e.g. the quality and corrections of the arterial input function [20, 21], variations in cardiac output [22] or variations in ctHb or blood gas levels (see later).

Other research groups have quantified blood flow of different tumour types of the brain with ^15^O-H_2_O PET [23]. Meningiomas were listed with a mean CBF of 57 ± 22 ml min^−1^ 100 g^−1^, which corresponds well to our patient with grade I meningioma (patient 6), who had a tumour CBF of 45.9 ml min^−1^ 100 ml^−1^. For the neuroepithelial tumours, results are reported with more variation [24–26], and rCBF varies greatly between tumours. In our study, data confirm that this is even true within the tumours, regardless of grade [27]. This is reflected in the sometimes large standard deviation of the mean CBF measured by PET perfusion (Table 3), ranging from 11 to 58 %. Also, contralateral grey matter perfusion values were generally lower than mean grey matter perfusion values measured on healthy subjects in PET, 34.9 ± 8.8 and 48.7 ± 5.0 ml min^−1^ 100 ml^−1^, respectively [10], which confirms data from Mineura et al. that grey matter perfusion can be suppressed in patients with brain tumours, even on the contralateral, healthy side [25].

In a study of the clinical applicability of CBF estimated with PCT in patients with gliomas, the normalized values of CBV correlated with tumour grade [19]. Their normalized CBF values with PCT (high grade CBF 2.7 ± 0.5, low grade 1.1 ± 0.4) compare well with our values (Table 4).

Evaluating the relative distribution between rCBF values, there was generally speaking a good match between both PET and PCT images in patients with low-grade glioma (Fig. 1) as well as between PET and PCT rCBF values. For high-grade glioma, however, there was a mismatch with rCBF values in the tumour clearly above the values in grey matter in PCT and below grey matter in PET (Table 4). Furthermore, one patient with glioblastoma multiforme had a hyperperfused area on both repeated rCBF PET scans anterior to the tumour situated in normally appearing cortex above the tumour oedema. This was not visualized in the subsequent PCT performed a few hours later and thus constitutes an obvious mismatch (patient 7, Fig. 2). We do not, however, believe this to be angiogenic activity in malignant tissue, as it would be unlikely that this relatively large area would not be apparent on MRI. More likely it is hyperperfusion, secondary to synchronous neuronal activity linked to a focal non-convulsive epileptic seizure that subsequently resolved prior to PCT. There were no obvious clinical signs of a seizure, but it is a well-known pitfall in the evaluation of ^18^F-fluorodeoxyglucose (FDG) PET scans of brain tumours, and it can be expected to pose a similar problem when identifying angiogenically active tumour tissue with PCT or MRI.

There is evidence to suggest that high angiogenic activity in itself, measured by rCBF with MRI, has predictive information of tumour progression or death independent from tumour grade [18]. The patient in our series with the highest tumour perfusion on both PET and PCT had in fact a low-grade astrocytoma (patient 9, Table 1). In another patient with a low-grade glioma (patient 1, Fig. 1) we found a heterogeneous distribution of rCBF values including hyperperfused tumour tissue. Thus, high angiogenic activity in itself is not a trait reserved only for high-grade tumours, and these patients would be false positives if grading was based on rCBF alone. Similar findings have been presented previously [18–28]. Interestingly, we found a significant method-dependent difference regarding the ability to grade tumours based on rCBF. Thus, there was a significant difference between tumour grade measuring rCBF with PCT, but not with PET. These results match the data obtained using steady-state susceptibility contrast-enhanced MRI (ssCE-MRI). With this MRI sequence it is possible to preferentially sensitize the rCBV measurements toward either all tumour vessel components or selectively the microvascular segments (<30 μm) using either gradient-echo echo-planar imaging (GE-EPI) or spin-echo echo-planar imaging (SE-EPI), respectively [29]. No significant difference was found between high- and low-grade gliomas using SE-EPI, while this was the case for GE-EPI [29–31]. It has been suggested that the sizes of vessels within a tumour vary with the aggressiveness and angiogenic activity of the tumour [32]. Thus, rCBF PET is primarily linked to the capillary level for which rCBV SE-EPI would also be a surrogate MRI parameter. So performing measurements of the capillary function does not appear as efficient in glioma grading, but these are the parameters most likely to portray the tumour microenvironment, not least with respect to the exchange of solutes, nutrients and chemotherapeutics.

On the other hand, PCT and GE-EPI that embrace all, even nonfunctional, vascular segments might add value in tumour grading [4–31] and offer a parameter to monitor the effects of anti-angiogenic therapies [33, 34], but cannot be used to examine the functional level of the tumour. These hypotheses, however, need to be validated by a direct comparison between techniques.

As an integrated part of the validation between PCT and PET, various error sources need to be considered. PET and PCT were performed within 1–4 h on the same day and always with PET performed before PCT for practical reasons. We do not have to consider a day-to-day measurement variance pertaining to variations in e.g. antiepileptic or prednisone drug concentration. However, an order effect cannot be excluded. The arterial blood gas analysis (Table 2) showed a relatively hypocapnic status with a decrease of 4 mmHg in PCT compared to PET suggesting slight spontaneous hyperventilation, which can probably be ascribed to an emotional response [35] in reaction to procedures relating to the PCT scanning technique itself (gantry noise/contrast injection). Thus, the technique might have been a more profound factor than scan order. Indeed, there is evidence that PCT systematically affects the respiratory pattern: In a previous independent study in healthy subjects we found signs of significant hypocapnia during PCT [10]. The vasoreactivity in PCT has not been investigated systematically in humans, but in PET a decrease in *P*_a_CO_2_ of 4 mmHg would lower the rCBF in grey matter in the order of 8 % [36] and an effect of the similar order in white matter [37]. In animal models using implanted carcinoma cells, the tumour tissue perfusion using PCT does respond to changes in *P*_a_CO_2_ level [38], albeit influenced by the anaesthesia used [38]. Furthermore, there are indications from MRI studies of implanted gliomas in the rat brain that cerebral blood vessels derived from tumour angiogenesis do retain reactivity to CO_2_, however, with an augmented response to hypocapnia compared to normal grey matter [39]. Whether these findings can be transferred to de novo gliomas in humans is not known. A similar physiology would lead not only to a decrease in tumour tissue perfusion, but also a decrease in the perfusion ratio of tumour to grey or white matter.

An important factor to explain the quantitative and qualitative difference between PCT and ^15^O-H_2_O PET adheres to the fundamental differences between a CT non-diffusible intravascular iodinated medium and a PET freely diffusible tissue tracer. “Perfusion” refers to blood flow per unit functional tissue mass. This would correspond to the attenuation signal that in PCT derives from capillaries. However, because of the inherent limitations of resolution the capillary signal cannot be discriminated from flow in larger vascular structures up- or downstream from the capillary level. These structures are not functional to the tumour tissue, in the sense that there is no exchange of nutrients, medication or tumour-derived humoral factors etc. across the vessel walls, and their presence in the imaging field will increase perfusion measurements erroneously. Adding to the difficulties is the known chaotic and bizarre architecture of blood vessels produced within tumours by chronically activated angiogenesis and an unbalanced mix of proangiogenic signals. Tumour neovasculature is characterized by precocious capillary sprouting, convoluted and excessive vessel branching, distorted and enlarged vessels, erratic blood flow, micro-haemorrhaging, leakiness and arteriovenous shunts [40]. Thus, even flow at the capillary level needs not be functional. The freely diffusible PET tissue tracer, however, does also have its limitations pertaining to the heterogeneous tumour tissue composition itself [41]. In fact the term “multiforme” in glioblastoma multiforme refers exactly to its heterogeneous macroscopic morphological presentation, with areas that are either cystic, white and firm, yellow and necrotic or red and haemorrhagic. There will indisputably be cystic and necrotic tumour compartments that will not be available for free exchange with ^15^O-labelled water. So although the rCBF PCT and the PET ROIs are identical and thus influenced equally by the tumour tissue heterogeneity, a purely intravascular tracer will overestimate perfusion because of nonfunctional vascular structures, while a freely diffusible tissue tracer will underestimate perfusion because nonfunctioning tissue such as cysts are interpreted as tumour tissue.

In conclusion, our study on gliomatous tumour rCBF determined by PCT compared to the gold standard ^15^O-H_2_O PET showed that PCT could not be used for absolute measurements of tumour blood flow.
